# Erectile dysfunction in primary care: practice patterns, barriers, and opportunities—narrative review

**DOI:** 10.1093/sexmed/qfag033

**Published:** 2026-06-01

**Authors:** Hossein Saadat, Michael L Pianezza, Dean Elterman

**Affiliations:** Division of Urology, Department of Surgical Subspecialties, Northern Ontario School of Medicine, Sudbury, ON P3E 5J1, Canada; Division of Urology, Department of Surgical Subspecialties, Northern Ontario School of Medicine, Sudbury, ON P3E 5J1, Canada; Division of Urology, University of Toronto, Toronto, ON M5T 2S8, Canada

**Keywords:** erectile dysfunction, primary health care, practice patterns, communication barriers, sexual health

## Abstract

**Introduction:**

Erectile dysfunction (ED) is a common but often underdiagnosed and undertreated condition. It affects 20% to 57.8% of men, with prevalence increasing with age. Despite established guidelines, inconsistencies in assessment and management persist in primary care.

**Objectives:**

To assess whether primary care ED management aligns with guidelines or reveals gaps causing underdiagnosis and undertreatment.

**Methods:**

Comprehensive literature search was conducted in PubMed, MEDLINE, TRIP, and Google Scholar for studies published between January 2001 and December 2024. Search terms included: ED, primary care, family physician, practice patterns, barriers, and management. Inclusion criteria were: studies focusing on primary healthcare providers’ practice patterns in the assessment and management of ED; studies examining barriers faced by primary care providers; and studies involving specialized care if findings were transferable to primary care. Review articles, case reports and very small case series (<10 participants) were excluded. Data were extracted on participant characteristics, management strategies, practitioner attitudes, comfort levels, and barriers to care. Methodological quality was assessed using critical appraisal principles.

**Results:**

Eleven studies met criteria: Seven cross-sectional surveys, two qualitative focus groups and two referral audits. Active discussion of ED occurred in 10% to 38% of encounters. Key barriers included limited training, time constraints, embarrassment, and unclear clinical roles. Standardized tools/questionnaires were under-utilized. Referral audits showed 26% to 37% of patients could have been managed in primary care. Evidence demonstrated low confidence, knowledge gaps and inconsistent adherence to guidelines.

**Conclusion:**

ED is common but often under-assessed and under-managed in primary care. Studies show limited proactive patient–provider communication, inconsistent diagnostic approaches, poor adherence to guidelines, and frequent premature referrals. Failure to initiate discussions about ED further contributes to underdiagnosis and delayed recognition of related conditions such as cardiovascular and metabolic diseases.

## Introduction

The prevalence of erectile dysfunction (ED) is reported to range from 20% to 57.8% depending on the questionnaire used for assessment.^1^^,^^2^ The risk of experiencing ED in men between 60 and 69 years of age is about 40% and there is a trend of increasing prevalence with advancing age.^3^

While ED can affect interpersonal relationships, quality of life and psychological well-being, it is also increasingly recognized as predictor or early marker for metabolic and systemic disease increasing the risk of cardiovascular disease and all-cause mortality.^4^

Several guidelines are available for the assessment and management of ED. There are however significant inconsistencies in the adoption of these management strategies. This has resulted in the undertreatment of ED in up to 70% of men affected with ED.^1^^,^^2^^,^^5^^,^^6^

A Canadian survey of 3009 adults found that 75% of men with ED had never discussed the issue with a healthcare provider (HCP). Among those who did, 29% were dissatisfied with the encounter, often citing that the concern was not taken seriously or that the clinician lacked adequate knowledge. The same study showed that subjective impressions of men’s sexual function substantially underestimated the true prevalence of ED. Self-reported rates of ED was only 11% for men or their partners, while rates assessed using the Sexual Health Inventory for Men were 22.3% and 26.7%, respectively.^7^ International data are similar: in a survey across six countries (United States, France, Germany, Italy, Spain, and the United Kingdom), 53% of men with ED had not sought treatment^8^ and rates in Denmark ranged from 62.4% to 68.2%.^9^^,^^10^ Contributing factors to low treatment-seeking behavior included embarrassment, low libido, limited time, socioeconomic barriers and both younger and older age groups.^8-10^ These findings highlight the critical role of primary healthcare providers in proactively assessing ED rather than relying on patient self-reporting alone.^10^

Platano et al.^11^ highlighted that psychosocial context plays a critical role in sexual history taking and that integrating psychological and relational components is essential when primary care providers address ED as part of a broader sexual health framework.

Considering the multifactorial nature of ED, a multidisciplinary approach by psychologists, psychiatrists, cardiologists, gynecologists and urologists is the optimal way for the assessment and management of this condition.^1^^,^^12^^,^^13^ Although a facility or program that offers this combined approach is ideal, establishing or even finding such a setup is often challenging.

This broader perspective underscores the importance of early detection in primary care. Given their ongoing relationships with patients and focus on preventive care, primary care providers are well positioned to identify ED and use it as an opportunity to assess cardiovascular and metabolic risk along with psychosocial factors.

This offers a valuable opportunity to initiate a more comprehensive approach to patient care, including lifestyle modification, risk factor optimization, and early intervention for chronic disease. Framing ED in this way shifts it from being solely a quality-of-life issue to a clinically meaningful marker that can support earlier diagnosis and improve long-term health outcomes.

This literature review with narrative synthesis evaluates ED management in primary care and aims to address the following question: Do the practice patterns of primary care providers in ED care reflect adherence to established guidelines, or do they reveal systematic gaps in assessment and management that contribute to underdiagnosis, undertreatment, and premature referral?

## Methods

**Figure 1:**
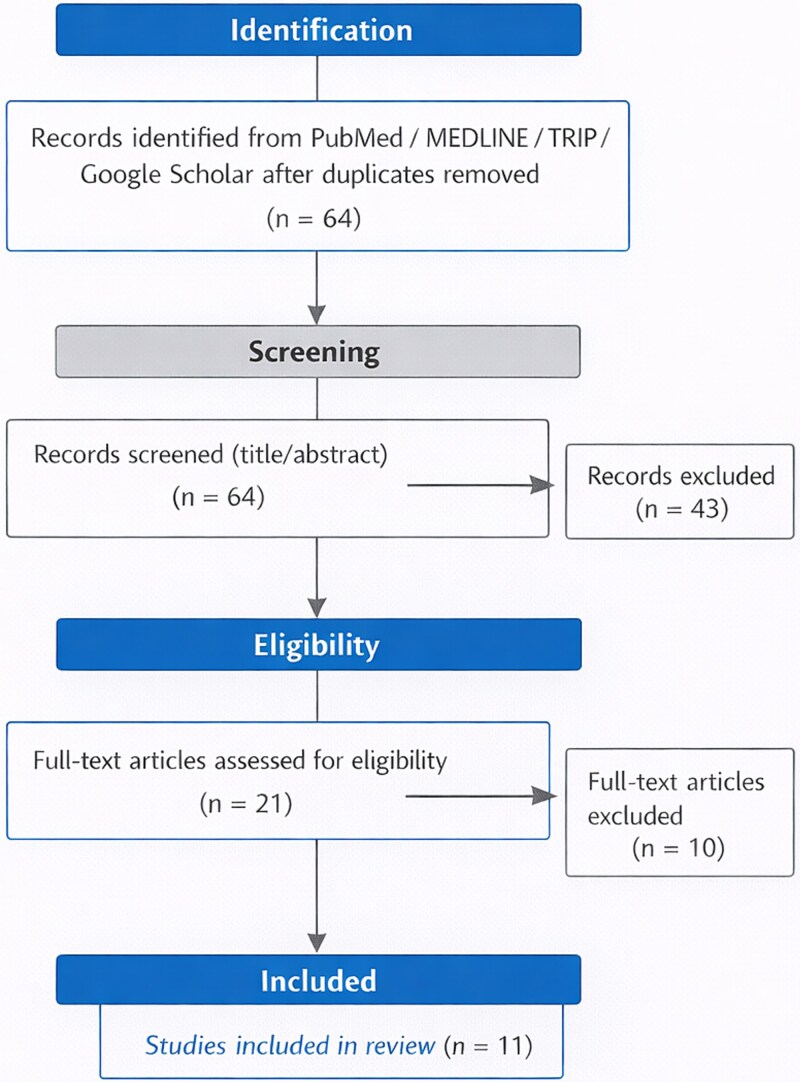
Flow diagram summarizing the study selection process.

Literature search for studies published between 2001 and December 2024 in PubMed, Medline and sources of gray literature such as TRIP and Google Scholar was performed. Search terms combined controlled vocabulary and free-text keywords: *erectile dysfunction*, *primary care*, *family physician*, *practice patterns*, *barriers*, and *management* (Figure 1). This study is a narrative review using a structured literature search and thematic synthesis. No specialized software or devices were used for data analysis.

### Inclusion criteria

- Articles focusing on primary healthcare providers and their practice patterns regarding the assessment and management of ED.
- Articles focusing on the barriers faced by primary healthcare providers in the assessment and management of ED.
- Articles focusing on specialized care if they provide transferable insights to primary care.

### Exclusion criteria

- Review articles.
- Case reports
- Very small case series (<10 participants)

### Study selection

No language filters were applied initially; non-English studies were excluded only if full-text translation was not accessible. Titles and abstracts were screened. Full-text articles were then evaluated for eligibility using the inclusion/exclusion criteria.

### Data extraction

A standardized data extraction form was used to capture:

- Study characteristics (year, country, design, sample size)
- Participant type (physicians, nurses, patients)
- Assessment and management practices
- Provider knowledge, comfort, and attitudes
- Patient-related barriers to seeking or engaging in ED care
- Reported structural, cultural, or system-level barriers
- Diagnostic and therapeutic strategies used prior to referral
- Key outcomes and conclusions

### Data synthesis

Given variability in study design, populations, and outcomes, a narrative synthesis approach was employed. Studies were grouped thematically into:

- Provider practice patterns
- Provider knowledge, attitudes, and comfort
- Patient-reported barriers to care
- System-level and structural constraints
- Primary-care management before referral

## Results

Eleven studies met the inclusion criteria. Table 1 summarizes practice patterns/confidence while Table 2 outlines barriers to ED management in primary care. The overall strength of evidence is moderate. Most studies were cross-sectional surveys,^14-20^ providing quantitative data on physician knowledge, attitudes and comfort levels. Two qualitative studies^23^^,^^24^ used focus group discussions to explore physician experiences and perceived barriers in managing ED. These studies offered valuable real-world insights but limited generalizability due to small sample sizes (*n* = 28 and *n* = 22).

**Table 1:** Practice patterns for assessment and management of ED in primary care.

	**Healthcare provider type**	**Patient demographics**	**Attitude, Knowledge, Comfort level**
S. D. Rutchik et al. (2001)^14^	Family Physicians	Diverse clinical contexts	Only (15%) routinely inquiring about ED in patients older than 40 years old. Clear discrepancy between confidence in diagnosis & treatment.
Platano (2008)^15^	General Practitioners and Urologists		Only 10.4% of GPs actively ask about male sexual dysfunction moderate or lower confidence rates for discussing and treating ED (44% and 60% of GPs)
Alarcão et al. (2012)^16^	General Practitioners		Lowof confidence rates (38.9% and 56.7% in male vs female GPs)
Ribeiro et al. (2014)^17^	General Practitioners		Routine sexual history taking and systematic consultation of established guidelines were uncommon among general practitioners.
Dyer et al. (2019)^18^	Primary and Secondary Healthcare Providers	Prostate cancer survivors	Limited communication between HCPs and patients. Failures to initiate discussions. Lack of consensus over roles and responsibilities among HCPs.
Tay et al. (2019)^19^	General Practitioners	Diverse clinical contexts	Low confidence in screening ED
Farajallah et al. (2024)^20^	General Practitioners	Diverse clinical contexts	Physicians rarely initiate discussions about ED. Only 57.6% prescribed a PDE5 inhibitor
Morgado et al, (2019)^21^	General Practitioners	Diverse clinical contexts in patients with ED referred to urologist	High rate of misdiagnosis Low treatment rates before referral Low rates of recommending lifestyle modifications
Iqbal et. Al. (2024)^22^	Primary healthcare providers	Diverse clinical contexts in patients with ED referred to urologist	Low rates of laboratory tests and cardiovascular risk assessment Low treatment rates before referral Low rates of recommending lifestyle modifications

**Table 2:** Barriers to ED management in primary care.

Category	Barrier	Source studies
Patient-related	Embarrassment, reluctance	^23^ ^,^ ^24^
Provider-related	Lack of training, low confidence or comfort level	^14-16^ ^,^ ^19^^,^ ^20^^,^ ^23^^,^ ^24^
System-related	Time constraints	^16^ ^,^ ^19^^,^ ^23^^,^ ^24^
Communication	Failure to initiate discussion	^14^ ^,^ ^15^^,^ ^17^^,^ ^18^^,^ ^20^^,^ ^23^^,^ ^24^
Treatment-related	Cost, access limitations	^23^ ^,^ ^24^

Two additional studies reviewed patients referred to urology clinics to evaluate care delivered in primary care before referral.^21^^,^^22^ The study by Morgado et al. included 115 patients and applied clear inclusion criteria with standardized assessment tools, including the International Index of Erectile Function (IIEF-5). Its analysis was strengthened by detailed stratification of cardiovascular risk factors and medication profiles, offering valuable insight into comorbidity and pharmacologic management. However the small sample size and single-center design limited external validity.^21^ Similarly, Iqbal et al. conducted a retrospective review of 148 men referred to a dedicated ED clinic in the United Kingdom. This study benefited from structured data collection and detailed outcome reporting. However the retrospective design of the study, its reliance on general practitioner (GP) documentation, small sample and single-center scope made it susceptible to selection and reporting bias. This limited the generalizability of the study.^22^

The following section outlines the principal findings and contextual details from the included studies.

### Details of included studies

In a survey of 85 family practitioners regarding their routines in assessing and managing ED, only 15% routinely inquired about ED during encounters with men over 40 years old. This percentage increased to just 51% when patients had risk factors.^14^ While 82% of respondents felt comfortable or somewhat comfortable diagnosing ED, only 54% expressed comfort with prescribing Sildenafil despite it being the most commonly used treatment. Comfort level with the vacuum erection device, intraurethral suppositories and intracavernosal injections were even lower at 15%, 10% and 2% respectively.

A study conducted in Switzerland by Platano (2008) highlighted the very limited frequency of structured sexual history taking in men, even among physicians routinely managing sexual dysfunction. Only 10.4% of GPs actively asked about sexual dysfunction. Low confidence rates for discussing and treating ED were reported. These findings reinforce that proactive sexual health discussion rarely occurs unless initiated by symptoms, underscoring the importance of encouraging routine inquiry in primary care.^15^

In a cross-sectional studies from Lisbon, using structured questionnaires answered by 50 GPs, Alarcão et al.^16^ identified that general practitioners frequently reported limited training, lack of confidence and time constraints as major barriers to ED assessment and management. Lack of confidence was reported by 38.9% of male GPs and 56.7% of female GPs. Initiating a conversation about ED was found to be affected by self-perceived competence, training and consultation of guidelines, years of practice, and average number of medical appointments per week addressing sexual dysfunction.

Ribeiro et al. in a cross-sectional study conducted in Lisbon, reported that only 15% of general practitioners (GPs) proactively initiated a discussion regarding sexual dysfunction, most commonly when underlying medical conditions such as diabetes were present. Furthermore, sexual dysfunction was included as part of routine history taking in only 22% of those who initiated such discussions. Routine sexual history taking and systematic consultation of established guidelines were uncommon among GPs; only a minority consistently explored sexual health or applied formal diagnostic frameworks, which may contribute to variability and inconsistency in clinical practice.^17^

A UK survey of 546 men following prostate cancer treatment with ED found that discussion of erectile function was infrequent: only 10% were asked about it before treatment and 29% were never asked afterward. Standardized tools such as Sexual Health Inventory for Men (SHIM) or IIEF were used in fewer than 10% of cases pre-treatment and 23% post-treatment. Many physicians viewed ED as a low priority problem unless raised by the patient. Although most providers acknowledged that GPs play a key role in ED care, 38% never and 29% rarely initiated such discussions. Access to advanced therapies remained limited, with 42%–64% of GPs reporting restricted availability of intracavernosal injections, vacuum devices, or penile prostheses. This survey demonstrated that HCPs had different perspectives regarding their roles and responsibilities in the management of ED. General practitioners identified their role as equally important as urologists for initiating ED management. Nurse practitioners however ranked GPs as second only to urologists. Both general and nurse practitioners agreed that GPs play a key role in monitoring ED management.^18^

A cross-sectional study across 11 primary care clinics in Malaysia assessed healthcare provider knowledge, attitudes, confidence and barriers regarding ED screening.^19^ Among the 77 participants who comprised of doctors, medical assistants and nurses, 67.5% had previously performed ED screening with a median of 10 patients screened in the past year (IQR 18). Only 20.8% however felt confident in conducting ED screening. Lack of training, limited consultation time and patient refusal were frequently identified as barriers. However only lack of training showed a statistically significant impact. Notably knowledge scores related to the types of ED and assessment tools were below the median interquartile range in over 71% of participants. This finding indicated substantial knowledge deficiencies. All participants agreed that ED is an important health issue that should be treated and 97.4% recognized the benefits of ED screening.

A recent survey of general practitioners in France revealed that the majority of general practitioners (80.2%) discussed ED only when initiated by the patient with just 19.8% raising the topic themselves. This typically occurred during treatment reassessment, initial consultation or post-cardiovascular events. Only 57.6% prescribed a PDE5 inhibitor as first-line treatment while many opted for reassurance or referral. Few GPs followed national guidelines and over 70% cited lack of medical knowledge as the main barrier. Most GPs felt poorly informed about ED treatments especially beyond PDE5Is. Lower consultation frequency was also linked to lower confidence in discussing treatment options.^20^

Factors affecting ED management were addressed in two qualitative focus group discussions with general practitioners (GPs) in Singapore.^23^^,^^24^ The following were highlighted as major barriers in the assessment and management of ED:

**1. Patient-Related Barriers**

- Reluctance in disclosing symptom
- Overwhelming emotional outbursts during discussions
- Preference for a quick fix
- Cultural embarrassment barriers
- Complex patient demographics
- Unrealistic medication expectations
- Avoidance of regular GP

**2. Medication-Related Barriers**

- High medication cost
- Side effect concerns
- Unregulated drug access

**3. Physician-Related Barriers**

- Passive, reactive patient-initiated approach
- ED under-recognized as a medical condition
- Female GPs less likely to initiate
- Low confidence and limited GP training
- Time pressure and patient volume constraints
- Fear of damaging patient rapport
- Bulk purchase and low profit barriers
- Moral and social prescribing concerns

A high rate of misdiagnosis (22.3%) was reported in a study of 115 patients referred to a urology clinic in Portugal. The most common missed diagnoses at the primary care level included premature ejaculation, Peyronie’s disease and decreased libido. Lifestyle modification was recommended to only 26% of patients and PDE5 inhibitors were prescribed to just 30% prior to referral.^21^

In another study of patients referred to a urology clinic in the UK, 89.9% had not received counseling on lifestyle changes and 61.5% had not undergone the necessary laboratory or cardiovascular assessments. Notably over 28% of these cases could have been effectively managed in primary care through appropriate initiation or adjustment of PDE5 inhibitors. An additional 6.7% of patients would have benefited from psychosexual and lifestyle counseling.^22^

## Discussion

Overall, there is limited evidence describing practice patterns and barriers related to the diagnosis and management of ED in primary care. Across the included studies, considerable variability was observed in both assessment and management practices among primary care physicians.

Taken together, communication between patients and primary healthcare providers (HCPs) regarding ED remains suboptimal. Discussion rates were reported between 15% and 38%, and only 25% in Canada, suggesting that many men with ED remain undiagnosed and untreated. This communication gap may contribute to delayed recognition of underlying cardiovascular or neurological disorders that can negatively affect the quality of life of patients and their partners.

Key contributors to this gap include discomfort and lack of clarity regarding the roles and responsibilities of primary HCPs in ED care and reluctance to initiate sexual-health discussions. This hesitation often arises from the perceived complexity of ED, uncertainty surrounding management and limited training or available resources. Although most physicians reported feeling comfortable diagnosing ED, fewer than 60% were confident prescribing phosphodiesterase type 5 inhibitors. Confidence was substantially lower for other therapeutic options such as vacuum erection devices, intraurethral suppositories or intracavernosal injections with comfort levels of 15% or less.

Studies demonstrate a strong correlation between provider knowledge and clinical practice patterns, underscoring the need for enhanced education.^16^^,^^25^ Addressing these barriers requires targeted investment in professional development and digital tools. Decision-support algorithms and artificial-intelligence–based screening aids could streamline ED assessment. In addition, small-group workshops and simulation-based training may improve physician confidence in managing ED with oral therapies or basic procedures such as intracavernosal injections.^26^^,^^27^ Such efforts could promote more proactive, patient-centered care, including routine use of standardized ED screening tools in primary care. Ribeiro et al. further demonstrated that consultation of guidelines and adequate appointment time were positively associated with active exploration of sexual dysfunction. Physicians who accessed and applied guidelines were significantly more likely to proactively discuss ED. This reinforces the importance of training and structured resources in improving ED care in primary practice.^17^

Literature shows low rates of treatment-seeking behavior resulting from various factors such as stigma, socioeconomic factors, or cultural barriers.^8-10^

Due to the longitudinal relationships with patients primary healthcare providers can have a unique position to promote routine screening for ED and possible underlying metabolic and cardiovascular conditions. The use of validated tools such as the IIEF or SHIM, combined with guideline-based management, can improve diagnostic accuracy and treatment outcomes in terms of psychosocial satisfaction, and management of underlying comorbidities.^1^^,^^2^^,^^4^^,^^5^^,^^13^

Including partner in the process of assessments and management of ED, can promote treatment seeking behavior and adherence to treatment.^28^

This review has several limitations. The number of included studies was small and heterogeneous in design, limiting generalizability. Most studies were cross-sectional surveys with potential response and selection bias. The inclusion of qualitative and retrospective studies further limits comparability. Publication bias may also be present. In addition, variability in definitions and outcome measures across studies restricted the ability to perform quantitative synthesis. Finally, as a narrative review, this study may be subject to selection bias despite a structured search strategy.

## Conclusion

ED remains under assessed and undermanaged in primary care, despite its high prevalence and established links to overall health. Across the available literature, variability in diagnostic practices, limited adherence to guidelines and premature referrals to secondary care were consistently observed. Communication between patients and healthcare providers remains low, contributing to underdiagnosis and delayed identification of associated comorbidities such as cardiovascular disease and metabolic syndrome.

These findings highlight the need for targeted strategies to strengthen ED management within primary care. Regional barriers in the assessment and management of ED should be identified and addressed through direct engagement with healthcare providers. Based on these identified barriers; decision-aid tools, educational workshops, and simulation-based training could be developed and implemented to enhance provider confidence, promote guideline adherence and encourage proactive communication about sexual health. Use of standardized intake forms, pre-visit questionnaires, digital tools, and artificial intelligence can promote screening, mitigate time-related barriers, improve confidence in the assessment and management of ED, and facilitate more proactive enquiry by primary healthcare providers. Ultimately, improving awareness, diagnostic accuracy and treatment confidence among primary care clinicians will help ensure earlier identification, optimized management and better quality of life for men affected by ED and their partners.
